# Latent and Active Tuberculosis: Evaluation of Injecting Drug Users

**DOI:** 10.5812/ircmj.6283

**Published:** 2013-09-05

**Authors:** Mojgan Mamani, Mohammad Mahdi Majzoobi, Saadat Torabian, Ronak Mihan, Kamyab Alizadeh

**Affiliations:** 1 Department of Infectious Diseases, Hamedan University of Medical Sciences, Hamedan, IR Iran; 2 Department of Social Medicine, Hamedan University of Medical Sciences, Hamedan, IR Iran; 3 Hamedan University of Medical Sciences, Hamedan, IR Iran

**Keywords:** Latent Tuberculosis, Drug Users, Tuberculin

## Abstract

**Background:**

There is a high risk of tuberculosis (TB) infection among injecting drug users (IDUs).

**Objectives:**

This study aimed to determine the frequency of latent and active TB infection among IDUs.

**Materials and Methods:**

In a cross-sectional study between 2008 and 2009, IDUs referred to the methadone maintenance treatment (MMT) centers in Hamedan-Iran, undergone tuberculin skin test (PPD; purified protein derivative) were recruited. The participants with positive results for PPD test (> 5 mm and > 10 mm in HIV positive and negative cases), undergone other complementary procedures such as chest-X-ray and sputum smear test.

**Results:**

Overall, 268 IDUs between 18 and 70 (mean: 34.5 [8.2]) years were included in the study. PPD test had positive findings in 49 cases (18.3%). There was no significant difference of PPD positivity between HIV positive and negative participants (17.7% vs. 18.5%). An active TB was found among IDUs.

**Conclusions:**

The high prevalence of latent and active TB among IDUs indicates the need for TB screening tests among this population.

## 1. Introduction

Tuberculosis (TB) remains a major infectious disease and one of the most important causes of death in the world from many years ago. Although the rate of TB has decreased over the recent decades, it is still a serious health issue among some high risk populations. Drug users are one of these high risk groups with an increased prevalence and a high incidence for TB infection (1). This is due to higher rates of HIV infection, high levels of poverty, homelessness, and other negative social conditions among drug users (2). Risk of HIV among injecting drug users (IDUs) prepares the way for TB infection. In such condition, HIV is the most potent risk factor for the reactivation of Mycobacterium tuberculosis infection (3). Heroin and opium addiction in Iran has the most prevalence in the world and based on Razzaghi et al. study one fifth of 15 to 60 aged Iranians are drug users (4). The injection as the route of substance administration among drug users has increased more quickly in Iran during the past decade (4, 5). This increased number of IDUs has led to development of the consequence problems such as HIV infection and TB. Identification of TB cases and control the infection with proper prophylactic program should be the top issue for prevention of developing latent TB infection to active form among this high risk group (6).

Moreover, it is important to complete the treatment of active TB cases for prevention of transmission to others and development of drug-resistant TB infection. The high risk of HIV infection among IDUs causes this population to have an increased risk of reactivation of latent TB and an enhanced susceptibility to progression to active TB following new infection (7). The screening programs and preventive treatment for TB have played major roles in reducing TB infection among most populations of the world. These screening programs are generally performed by using the skin testing with purified protein derivative (PPD) tuberculin. PPD is often used for routine screening of high risk individuals; and patients with positive response to PPD test have annually 5 - 8% risk of TB infection and 30% or more for developing active TB. Studies in different countries show a variety range of PPD test results among populations (8).

## 2. Objectives

In this study we aimed to determine the frequency of latent and active TB infection among IDUs referred to the methadone maintenance treatment (MMT) centers in Hamedan, a city in the west of Iran.

## 3. Materials and Methods

This cross-sectional study was performed on 400 IDUs over a period of one year (March 2008 ‒ February 2009) in the MMT centers in Hamedan. The study protocol was performed in accordance with the declaration of Helsinki and subsequent revisions and approved by ethics committee at Hamedan University of Medical Sciences. A written informed consent was obtained from participants before entering the study. Demographic and personal characteristics of the patients, including age, sex, occupation, history of drug injection including age at the first injection experience, duration of injecting drug use, and type of substance use were obtained by interviewing the participants and recording in an information form. Any history of jailing was notified as well.

All participants underwent enzyme-linked immune-sorbent assay (ELISA) anti-HIV Ab testing for HIV infection, and if resulted positive, the infection was confirmed with Western blot. A PPD skin test was performed for participants by an infectious disease specialist and was read by the same investigator after 48 - 72 hours. Positive PPD determined as induration reaction more than 5 mm in HIV positive cases and more than 10 mm in HIV negative cases. A chest-X ray (CXR) was performed for all cases with positive results for PPD cases, and the result was defined as normal or abnormal. Three sputum specimens for detecting AFB (acid-fast bacilli) in smears of each individual were sent to the reference lab in Hamedan province. The data was analyzed by SPSS software version 16 (SPSS Inc., Chicago, IL, The USA), and chi-square and if necessary Fisher exact test, and Mann-Whitney U test were used to compare the variables. A P values < 0.05 was considered as statistically significant.

## 4. Results

Over a period of one year 268 IDUs were participated and completed the study. Table 1 shows the demographic data and drug use characteristics in the population. 

**Table 1.** Demographic Data and Personal Characteristics of Drug Use in Male and Female Participants

	Total ^a^	Men	Women	P value
**Number of participants, No. (%)**	268 (100)	240 (89.5)	28 (10.5)	
**Age, y**	34.5 (8.2)	34.3 (8.0)	35.8 (9.2)	0.24
**Current occupation**				
Unemployed	137 (51.1)	109 (45.4)	28 (100)	< 0.0001
**Types of drug**				
Heroin	194 (72.4)	182 (75.8)	12 (42.8)	
Crack	90 (33.6)	76 (31.7)	14 (50)	
Tamjizak	42 (15.7)			
Norjizak	15 (5.6)			
**First injection age, y (%)**	23.2 (7.6)	22.9 (6.8)	28.3 (9.8)	< 0.0001
**Duration of injection, y (%)**	6.3 (6.0)	6.3 (5.9)	4.4 (3.1)	0.23
**HIV, No (%)**	79 (29.5)	79 (32.9)	-	
**History of imprisonment**	224 (83.6)	221 (92.1)	3 (10.7)	< 0.0001

^a^ Values are mean standard deviation and numbers are in percentage.

There were 240 men (89.5%) and 28 women (10.5%) among the participants. The mean age of IDUs was 34.5 (8.2) (range: 18 - 70) years. There was no significant difference in the mean of age between men and women (P=0.24). Among all cases, 137 participants (51.1%) were unemployed including all women and 45.4% of men (109 cases). Totally, the most common substance used among IDUs was heroin (72.4%). While heroin was the most prevalent substance among men (75.8%), crack was the most common substance in women (50%). The first injection experience in 288 cases (85%) was between 15 and 30 years. The mean age of starting injection drug was 23.2 (7.6) (range: 9 - 57) years. There was a significant difference between men and women regarding the age of starting injection drug (22.3 [6.8] vs. 28.3 [9.8] in men and women, respectively; P < 0.0001). In total, the mean duration of injection drug use was 6.3 (6.0) (range: 1 - 35) years and ranged from 1 to 35 years. The duration of injection was one year in 20.9% of IDUs and more than 10 years in 14.6% of them. There was no significant difference between men and women in this regard (6.3 [5.9] and 4.4 [3.1] years in men and women, respectively; P = 0.23). HIV infection was seen in 79 cases (29.5%) which all of them were male.

Previous history of jailing was noted in 83.6% of participants and there was a significant difference between men and women (92.1% of men vs. 10.7% of women; P < 0.0001). Table 2 shows the comparison of variables between positive and negative PPD cases. Totally, 49 (18.3%) cases had a positive PPD test result which all of them were men (20.4% of men). 

**Table 2.** Comparison of Variables Between Positive and Negative Tuberculin Skin Test Cases

	PPD ^a^		P value
	+	‒	
**No. (%)**	49 (18.3)	219 (81.7)	
**Gender**			
Male	49 (20.4)	191 (79.6)	0.004
Female	‒	28 (100)	
**Age**	36.8 (9.0)	33.9 (7.9)	0.02
**Current occupation**			
Self-employed	20 (40.8)	65 (29.7)	0.4
Employed	1 (2)	4 (1.8)	
Unemployed	22 (44.9)	115 (52.5)	
Worker	6 (12.2)	35 (16)	
**Types of drug**			
Heroin	32 (65.3)	162 (74)	0.1
Crack	15 (30.6)	75 (34.2)	0.3
Tamjizak	7 (14.3)	35 (16)	0.4
Norjizak	2 (4.1)	13 (5.9)	0.09
**First injection age, No. (%)**	22.3 (8.0)	23.4 (7.4)	0.37
**Duration of injection, No (%)**	8.6 (8.0)	5.8 (5.1)	0.003
**HIV**			
(+) (n = 79)	14 (28.6)	65 (29.7)	1
(‒) (n=189)	35 (71.4)	154 (70.3)	
**History of imprisonment**			
(+) (n = 224)	45 (91.8)	179 (81.7)	0.09
(‒) (n = 44)	4 (8.2)	40 (18.3)	

^a^ Values are mean standard deviation and numbers are in percentage.

The mean age in PPD positive group was 36.8 (9.0) vs. 33.9 (7.9) years in PPD negative group and there was a significant difference between the two groups (P = 0.02).The mean duration of injection drug use in PPD positive group was 8.6 (8.0) years; while it was 5.8 (5.1) years in PPD negative group. There was a significant positive correlation between duration of injection drug use and positive PPD result (P = 0.003). The mean age at first drug injection in positive and negative PPD groups were 22.3 (8.0) and 23.4 (7.4) years, respectively. The mean age at first injection were not significantly different between positive and negative PPD groups (P = 0.37). Of 49 PPD positive cases, 13 cases (26.5%) had abnormal findings in CXR, mostly with a pattern of bronchiectasis or emphysema. Only one of these 13 cases had infiltration pattern in the right upper lobe which was diagnostic for TB; in addition to a positive sputum smear.

There was no significant difference between substance types regarding the frequency of positive PPD test results (P > 0.05). There were 14 HIV positive patients (28.6%) among PPD positive cases, while the rate of positive HIV cases among PPD negative results was 29.7% (65 cases). There was no significant difference in the rate of HIV patients between PPD positive and negative groups. Of 49 cases with a positive result for PPD test, 45 cases (91.8%) had a history of jailing. There was no significant difference in history of jailing between cases with or without PPD positive test (P = 0.09).

## 5. Discussion

In the present study, the prevalence of positive tuberculin skin test reaction among IDUs was 18.3%. Deiss et al. published a review study in 2009 which indicated that several researches had a range of 10% to 59% for latent TB among various cohorts of drug users (9). Although some in vitro studies indicated the damage of drug use on the immune system (10, 11), it is clear that drug use is along with some epidemiological and environmental factors such as homelessness, tobacco and alcohol use, history of prison, and poor health status which may contribute to the high prevalence of latent TB among drug users and dispose this population for TB infection (12-17).

Our study indicated that longer duration of injection drug use correlates with higher prevalence of positive TB skin test. Other studies had similar results to our finding regarding the duration of drug use (9). Also, these studies demonstrated that the prevalence of latent TB was more in older age, which is similar to our result. About 30% of our cases were HIV positive. Regarding HIV infection, the prevalence of positive PPD test was equal in HIV seropositive and seronegative persons (17.7% versus 18.5%). Selwyn et al. conducted a prospective study on 520 IDUs in the US and evaluated the risk of active TB among HIV positive patients (8). The positive PPD skin test in HIV positive patients was 23%, and in HIV negative cases was 20%. The rate of positive PPD was not different between the two groups. These results were similar to the present study. In contrast with these findings, Golub et al. (18) demonstrated that PPD positive result was seen in 16% vs. 39% of HIV positive and negative IDUs, respectively. The possible reason for these discrepancies can be explained by the differences between CD4+ counts in HIV infected patients which results different immunoresponse to PPD test. As Portu et al. (19) indicated that in HIV positive group there was a significant association between results of PPD test and CD4+ T-lymphocyte count. The rate of positive PPD test was similar in HIV seropositive and seronegative cases when the CD4+ count was equal or greater than 500 cells/mm3. However, in cases with lower CD4+ counts, the rate of positive PPD tests was lower in HIV positive patients compared to HIV negatives cases.

Our study found a case of active TB with a positive sputum smear and an atypical manifestation of TB in CXR which was reported and anti-TB treatment was started. This finding showed the importance of screening among IDUs for latent and active TB infection to prevent the possibility of infection transmission to others. An important limitation of our study was the diagnostic method of TB infection which was based on a positive PPD skin test among IDUs. This skin test has a low specificity due to cross reactivity with proteins present in other mycobacteria such as the Bacille Calmette Guerin (BCG) vaccine strain, M. avium complex organisms, and other non-TB mycobacteria (6, 20-22). Furthermore, the sensitivity of PPD skin test is reduced in HIV positive cases (6, 23, 24). Thus, the prevalence of TB among our HIV positive patients should be more than the present report.

In conclusion, the overall frequency of latent TB infection among IDUs in Hamedan was 18.3% which is a high rate. Since PPD results in HIV positive and negative cases were similar, latent and active TB infection screening is important in both of these groups. The presence of an active TB infection among our cases shows the importance of active TB screening among IDUs. While we do not know the PPD history of patients it is better to do PPD at the entrance to prison and recheck it every year. Further investigations with more cases in whole the country are suggested to detect latent and active TB among this high risk population. The studies can use novel screening tests such as "whole-blood interferon-gamma release assay" with higher sensitivity and specificity than PPD to reduce false positive and false negative results.
